# The association between parental migration and early childhood nutrition of left-behind children in rural China

**DOI:** 10.1186/s12889-020-8350-4

**Published:** 2020-02-18

**Authors:** Huifeng Shi, Jingxu Zhang, Yufeng Du, Chunxia Zhao, Xiaona Huang, Xiaoli Wang

**Affiliations:** 1 Department of Maternal and Child Health, School of Public Health, Peking University, 38 Xueyuan Road, Haidian District, Beijing, 100191 China; 2 Section of Health, Nutrition, and Water, Environment and Sanitation, UNICEF China, 12 Sanlitun Road, Chao Yang District, Beijing, 100600 China

**Keywords:** Parental migration, Left-behind children, Early childhood, Undernutrition, Anemia, Rural China

## Abstract

**Background:**

More than one-third of children under 3 years old are left behind at home due to parental migration in rural China, and we know very little about early childhood nutrition of left-behind children (LBC) because of the dearth of research. This study examined the association between parental migration and early childhood nutrition of LBC in rural China.

**Methods:**

We used repeated cross-sectional data of rural children aged 6–35 months who participated in two surveys in six counties of northern and southern China in 2013 and 2016 respectively. The length, weight, and hemoglobin concentration were measured by trained health-care workers blinded to parental migration status. Stunting, underweight, wasting, and anemia were identified with the standards recommended by WHO. Generalized linear regressions and multivariate logistic regressions were employed to explore the association between parental migration and these nutritional outcomes at each time point.

**Results:**

Two thousand three hundred thirty-six and 2210 children aged 6–35 months were enrolled in 2013 and 2016, respectively. The results show a reduction of the risks of stunting, underweight, and wasting from 2013 (16.4, 8.5, and 3.5%, respectively) to 2016 (12.1, 4.0, and 1.5%, respectively) but highlight a constantly and alarmingly high risk of anemia among these children (44.8% in 2013 and 43.8% in 2016). Children with migrant fathers performed as well as or better than those with non-migrants on these indicators. Children with migrant parents performed slightly worse in 2013, but equal or slightly superior in 2016 on these indicators compared with children with non-migrants and migrant fathers. Children aged 6–17 months with migrant parents had a significantly lower risk of anemia than those living with their mothers or with both parents (43.1% vs. 63.6% and 61.5 in 2013, and 42.5 vs. 60.1 and 66.2% in 2016), even after controlling for children’s sociodemographic characteristics.

**Conclusions:**

Parental migration may be not detrimental and even beneficial to early childhood nutrition of LBC in rural China. Continued nutritional support is needed for all rural children, especially interventions for preventing micronutrient deficiency. Programs for LBC are recommended to continue to focus on nutrition but pay more attention to other important health issues.

## Background

There are about 1 billion migrants globally [1]. In China, since the reform and opening-up in the 1980s, a large number of rural residents have migrated from their homes to cities in search of better employment opportunities. However, because of the unstable income, unfriendly settlement policies, and limited access to public services (such as education and health care) in host cities, most labor migrants leave their children at their homes in the countryside with another parent or other family members. According to the latest statistics, in 2015, 40.51 million rural children were left behind due to parental migration, accounting for 29.4% of all rural children and 15% of all children in China [2].

The nutritional status of left-behind children, as one of their most important health outcomes, has drawn a lot of attention from researchers and policy-makers. A growing body of research in this field over the last three decades has yielded conflicting results. Most studies found that left-behind children were more likely to be stunted, underweight, or wasted compared with other children [3–11], but others found that left-behind children performed similarly to, or even better than, non-left-behind children on these anthropometric indicators [12–15]. In addition, some studies identified a higher risk of anemia among left-behind than non-left-behind children [16, 17], but other work found no such difference [14]. A recent systematic review and meta-analysis of these studies done from 1994 to September 2018, 82% of which were conducted in China, found that, compared with non-left-behind children, left-behind children had a significantly increased risk of wasting and stunting, but a similar risk of underweight and anemia [18].

Further research is necessary for consideration of the confusing findings and the possible shift in the impact of parental migration on child nutrition with socioeconomic development. In addition, despite numerous studies existing, some notable gaps in our knowledge remain. Firstly, most studies on nutrition of left-behind children focused on school-age children and few included infants and young children [18]. The findings from school-age children cannot be simply generalized to younger children because of the considerable differences in growth trajectories and living environment. Studies have provided strong evidence that undernutrition in the first 3 years of life has a long-term negative effect on an individual’s health and development in subsequent childhood and adulthood, and intervention is problematic once this window has passed [19–21]. Therefore, further research is needed to take a clear picture of early childhood nutrition of left-behind children to allow policy-makers and health-care providers to optimize policy orientation and resource allocation for improvement of children’ well-being, particularly in rural China, where 38.1% of children under 3 years old experience separation from one or both parents due to parental migration [2]. Secondly, most previous studies did not determine the impacts of different parental migration statuses on child nutrition. The limited evidence available suggests that maternal or both-parental migration may be more detrimental to child nutrition than paternal migration [16, 22].

In this study, we examined the association between various parental migration status and the nutrition of children aged 6–35 months in rural China using repeated cross-sectional data. This study will enhance our understanding of the nutritional status of left-behind children and the need for special interventions in early childhood and, by providing evidence for identifying health-related policy priorities, will enable the development of cost-effective interventions to improve the well-being of these vulnerable populations.

## Methods

### Design and participants

We used repeated cross-sectional data comprising two surveys conducted in six counties of northern and southern China in 2013 and 2016, respectively. The first survey was conducted prior to an early childhood development program in six countries of two provinces from July to September 2013: Songtao, Liping, and Pan Counties in Guizhou Province, and Fenxi, Lin, and Fangshan Counties in Shanxi Province [23]. In each county, a clustered random sampling method was used to select villages that met the following criteria: reachable by car from the county capital, with ≥50 resident children under 3 years of age, and with a sufficient number of caregivers willing to participate in the survey. Finally, totals of 40 intervention villages and 43 control villages were included in the study. All children under 3 years old in these selected villages were eligible for participation in the survey. The second survey was conducted in the same villages from July to September 2016 by the same method, and the participants were another group of children under 3 years old at this survey time who were not included in the first survey. Finally, totals of 2953 and 2745 children under 3 years old were enrolled in 2013 and 2016, respectively. According to the number of eligible children (*n* = 4288) in 2013, the response rate was 68.9% in 2013 and 64.0% in 2016.

According to the research objective, our analysis did not include children under 6 months old (375 in 2013 and 335 in 2016) because they were not measured for hemoglobin (Hb) concentration. Among children aged 6–35 months who were measured for at least one nutritional outcome (length, weight or hemoglobin concentration) (2560 in 2013 and 2398 in 2016), we also excluded twins, single-parent children, and those with serious diseases or disabilities (186 in 2013 and 125 in 2016). In addition, we did not include children with only migrant mothers because of the small number of such children (38 in 2013 and 63 in 2016). Finally, this present study only used data of children aged 6–35 months with available nutritional outcomes and with non-migrant parents (NLBC), migrant fathers (FLBC), or both migrant parents (PLBC).

### Measure

Face-to-face interviews with caregivers were conducted by uniformly trained local health workers. Data were immediately input, saved, and transmitted to statisticians by means of an electronic questionnaire application, which had a basic logic and integrity-checking function to enable investigators to correct errors and supplement omissions in a timely manner.

#### Basic characteristics

The following sociodemographic characteristics of the children and their caregivers were collected: (a) child gender, age, preterm, and ethnicity; and (b) caregiver’s relationship to the child, gender, age, and educational attainment. Depression among caregivers was measured using Zung’s self-rating depression scale (ZSDS), which is validated and used worldwide and consists of 20 items representing depressive features with a total score ranging from 20 to 80 [24]. ZSDS was administered by trained interviewers, and a ZSDS score of ≥50 was defined as depression. Household economic status was measured by the number of the following household electrical appliances and vehicles owned: telephone, washing machine, refrigerator, and TV in 2013; and telephone, washing machine, refrigerator, TV, motorcycle, tricycle, and car in 2016. Low household economic status was defined as owning fewer than three and four of these items in 2013 and 2016, respectively.

Some children in Songtao, Liping, Fenxi, and Lin counties received the interventions of the early childhood development program from 2014. Therefore, two variables related to the interventions were measured and controlled for as confounding factors in the analysis of the 2016 survey data. The first variable is the frequency of consumption of Yingyangbao supplements (a daily intake package of soybean-based micronutrient fortified powders), which was classified as: never supplemented, ever supplemented but none in the past week, 0–6 packages in the past week, and ≥ 7 packages in the past week. The second is the frequency of nutritional consultations with health-care workers in the past 6 months, which was classified as: never, less than once per month, and one or more per month.

#### Child breastfeeding and dietary intakes

As recommended in *Indicators for Assessing Infant and Young Child Feeding Practices* by the World Health Organization (WHO) [25], a 24-h reported food recall was performed to assess breastfeeding and the dietary intake of the following seven food groups: (a) grains, roots and tubers, (b) legumes and nuts, (c) dairy products, (d) flesh foods (meat or fish), (e) eggs, (f) vitamin-A rich fruits and vegetables, and (g) other fruits and vegetables. Then the following indicators were calculated: (1) ever being breastfed, (2) duration of breastfeeding (only for children weaned from breastfeeding), (3) meal frequency (frequency of dairy, solid, semi-solid, and soft food intake during the previous day), and (4) dietary diversity (number of food groups children consumed during the previous day).

#### Nutritional outcomes

The length, weight, and peripheral blood Hb concentration of the children were measured using standard procedures by uniformly trained health-care workers who were blinded to the above interview information. Children were weighed twice in light clothes without shoes using an electronic weight scale with 0.01 kg accuracy. Their recumbent, barefooted, and bareheaded lengths were measured twice using a standard infant length scale with 0.1 cm accuracy. A third measurement was performed if the two measurements differed by 1.0 cm or more for length and 0.5 kg or more for weight. The Hb concentration of the children was measured using HemoCue201+ (HemoCue AB Inc.). The length-for-age Z score (LAZ), weight-for-age Z score (WAZ), and weight-for-length Z score (WLZ) were calculated according to the WHO Child Growth Standards and the corresponding score of < − 2 was used to define stunting, underweight and wasting, respectively [26]. According to the WHO guidelines, Hb concentration was adjusted for altitude and children with an altitude-adjusted Hb concentration of < 11 g/dL were considered to be anemic [27].

### Statistical analysis

To double validate any associations and take into account the effect of time, we analyzed the data of the two time points independently. Univariate analysis was first conducted to compare the basic characteristics and nutritional outcomes according to parental migration status by *t*-test and Mann-Whitney U test for continuous variables and the chi-squared test for categorical variables.

Generalized linear regressions were performed to estimate the adjusted mean differences (aMDs) with 95% confidence intervals (CIs) in LAZ, WAZ, and WLZ scores and Hb concentration according to parental migration status at each time point, after controlling for resident county, child gender, age, preterm and ethnicity in 2013, as well as additional two intervention variables in 2016. Household economic status and caregiver’s relationship to the child, gender, age, and educational attainment were not controlled for because they were severely collinear with parental migration status and were strongly considered as mediators of the effects of parental migration on child nutrition. For categorical nutritional outcomes including stunting, underweight, wasting, and anemia, multivariate logistic regressions were employed to calculate the adjusted odds ratios (aORs) and 95% CIs after controlling for the same covariates as above.

Similar multivariable adjusted analyses were also performed on Hb concentration and anemia for children aged 6–17 and 18–35 months, respectively, based on the result of the preliminary analysis that the differences in Hb concentration and the risk of anemia between children with different parental migration statuses varied before and after the age of 18 months.

Statistical analyses were performed using Statistical Package for the Social Sciences (SPSS) software 20.0 (SPSS, Inc., Chicago, IL). A two-tailed *p*-value of < 0.05 was taken to reflect statistical significance.

## Results

### Study population

In total, 4546 children aged 6–35 months (2336 in 2013 and 2210 in 2016) were included in this study. They consisted of 1285 and 1089 NLBC, 819 and 784 FLBC, and 232 and 337 PLBC in 2013 and 2016, respectively.

The characteristics of the children are presented in Table 1. In 2013 and 2016, NLBC and FLBC had similar median ages (19–20 months) but were 2–4 months younger than PLBC. The distribution of child gender and preterm birth did not differ significantly according to parental migration status.

**Table 1:** Characteristics of the children and caregivers

Characteristics	2013 survey			2016 survey		
	NLBC (*n* = 1285)	FLBC (*n* = 819)	PLBC (*n* = 232)	NLBC (*n* = 1089)	FLBC (*n* = 784)	PLBC (*n* = 337)
Resident county, n (%)						
Lin	181 (14.1)	110 (13.4) ^†^	14 (6.0) ^‡§^	150 (13.8)	98 (12.5) ^†^	21 (6.2) ^‡§^
Fenxi	157 (12.2)	242 (29.5)	7 (3.0)	113 (10.4)	239 (30.5)	71 (21.1)
Fangshan	229 (17.8)	170 (20.8)	17 (7.3)	191 (17.5)	114 (14.5)	20 (5.9)
Songtao	120 (9.3)	28 (3.4)	43 (18.5)	100 (9.2)	29 (3.7)	37 (11.0)
Liping	100 (7.8)	74 (9.0)	110 (47.4)	109 (10.0)	87 (11.1)	76 (22.6)
Pan	498 (38.8)	195 (23.8)	41 (17.7)	426 (39.1)	217 (27.7)	112 (33.2)
Boys, n (%)	727 (56.6)	467 (57.0)	121 (52.2)	601 (55.2)	440 (56.1)	169 (50.1)
Child age (months), median (25th, 75th)	20 (13, 27)	19 (11, 27)	23 (17, 28) ^‡§^	20 (12, 27)	20 (12, 27)	22 (18, 29) ^‡§^
Preterm children, n (%)	39 (3.0)	24 (2.9)	2 (0.9)	54 (5.0)	47 (6.0)	11 (3.3)
Minority ethnic of children, n (%)	435 (33.9)	199 (24.3) ^†^	167 (72.0) ^‡§^	394 (36.2)	205 (26.1) ^†^	163 (48.4) ^‡§^
Relationship of caregivers to the child, n (%)						
Parents	1243 (96.7)	776 (94.7)	0 (0.0) ^‡§^	1048 (96.2)	733 (93.5) ^†^	0 (0.0) ^‡§^
Grandparents	39 (3.0)	42 (5.1)	229 (98.7)	39 (3.6)	47 (6.0)	321 (95.3)
Others	3 (0.2)	1 (0.1)	3 (1.3)	2 (0.2)	4 (0.5)	16 (4.7)
Female gender of caregiver, n (%)	1035 (80.5)	809 (98.8) ^†^	187 (80.6) ^§^	938 (86.1)	762 (97.2) ^†^	279 (82.8) ^§^
Caregiver age (years), median (25th, 75th)	27 (24, 31)	26 (24, 30)	51 (48, 56) ^‡§^	27 (24, 32)	27 (24, 31)	52 (47, 56) ^‡§^
Education attainment of caregivers, n (%)						
High school or above	165 (12.9)	123 (15.0)	8 (3.4) ^‡§^	218 (20.0)	158 (20.2)	18 (5.3) ^‡§^
Middle school	694 (54.0)	462 (56.4)	42 (18.1)	560 (51.4)	423 (54.0)	87 (25.8)
Primary school	343 (26.7)	186 (22.7)	66 (28.4)	249 (22.9)	162 (20.7)	104 (30.9)
Illiteracy	83 (6.5)	48 (5.9)	116 (50.0)	62 (5.7)	41 (5.2)	128 (38.0)
Depression among caregivers ^a^, n (%)	485 (40.0)	291 (37.1)	117 (50.6) ^‡§^	388 (35.9)	249 (31.8)	128 (38.0) ^§^
Low household economic status ^b^, n (%)	180 (14.0)	79 (9.6) ^†^	44 (19.0) ^§^	157 (14.4)	122 (15.6)	89 (26.4) ^‡§^

*NLBC* non-left-behind children, *FLBC* left-behind children with migrant fathers, *PLBC* left-behind children with both migrant parents

^a^73 NLBC, 34 FLBC and 1 PLBC in 2013, and 9 NLBC and 2 FLBC in 2016 missed the information of depression among caregivers

^b^1 NLBC in 2013 and 1 FLBC in 2016 missed the information of household economic status

^†^ FLBC vs. NLBC *p* < 0.05; ^‡^ PLBC vs. NLBC *p* < 0.05; ^§^ PLBC vs. FLBC *p* < 0.05

The characteristics of the caregivers were similar in the two surveys. NLBC and FLBC were primarily cared for by their mothers. Their median age was 26–27 years and about 70% of them were educated to a middle school or higher level. More than 95% of PLBC were cared for by their grandparents (median age of around 50 years), and less than one-third of the caregivers were educated to a middle school or higher level (21.5% in 2013 and 31.1% in 2016). In 2013, the caregivers of PLBC had a significantly higher risk of depression than the caregivers of FLBC or NLBC (50.6% vs. 37.1 and 40.0%, *p* < 0.05). The risk of depression among the caregivers in 2016 was lower than that in 2013, and the prevalence of depression among the caregivers of NLBC, FLBC, and PLBC was 35.9, 31.8, and 38.0%, respectively (Table 1).

FLBC had a higher household economic status than NLBC in 2013, but a similar one in 2016. PLBC had a lower household economic status than NLBC and FLBC in both 2013 and 2016 (Table 1).

### Stunting, underweight and wasting

Table 2 shows the anthropometric outcomes of children with various parental migration statuses. From 2013 to 2016, the prevalence of stunting (NLBC: 19.0 to 13.4%, by 29.5%; FLBC: 11.8 to 8.9%, by 24.6%; PLBC: 18.6 to 15.4%, by 17.2%; Total: 16.4 to 12.1%, 26.2%), underweight (NLBC: 9.2 to 4.3%, by 53.3%; FLBC: 6.5 to 4.0%, by 38.5%; PLBC: 12.1 to 3.0%, by 75.2%; Total: 8.5 to 4.0%, by 52.9%) and wasting (NLBC: 3.3 to 1.6%, by 51.5%; FLBC: 3.3 to 1.8%, by 45.5%; PLBC: 5.2 to 0.6%, by 96.2%; Total: 3.5 to 1.5%, by 57.1%) decreased in all groups of children. Greater reductions in the risks of underweight and wasting were found, especially among PLBC.

**Table 2:** The anthropometric outcomes of children aged 6–35 months with various parental migration statuses

	Outcomes [mean (SD)/ n/N (%)] ^a^				Adjusted differences [aMD (95%CI) / aOR (95%CI)] ^b^		
	NLBC	FLBC	PLBC	Total	FLBC vs. NLBC	PLBC vs. NLBC	PLBC vs. FLBC
2013							
Length/height-for-age z-score	-0.89 (1.45)	−0.57 (1.33) ^†^	−1.07 (1.25) ^§^	− 0.80 (1.40)	**0.13 (0.01, 0.26)**	0.08 (− 0.13, 0.28)	− 0.02 (− 0.23, 0.19)
Stunting (<− 2 z-scores)	234/1231 (19.0%)	92/781 (11.8%) ^†^	42/226 (18.6%) ^§^	368/2238 (16.4%)	**0.73 (0.56, 0.96)**	0.79 (0.53, 1.19)	1.08 (0.70, 1.68)
Weight-for-age z-score	−0.46 (1.34)	−0.19 (1.24) ^†^	− 0.77 (1.29) ^‡§^	−0.40 (1.31)	0.11 (− 0.01, 0.22)	0.07 (− 0.12, 0.25)	−0.02 (− 0.22, 0.18)
Underweight (<− 2 z-scores)	114/1245 (9.2%)	52/797 (6.5%) ^†^	28/232 (12.1%) ^§^	194/2274 (8.5%)	0.77 (0.54, 1.09)	1.14 (0.69, 1.87)	1.49 (0.86, 2.55)
Weight-for-length/height z-score	0.10 (1.13)	0.19 (1.18)	−0.24 (1.15) ^‡§^	0.09 (1.16)	0.02 (−0.08, 0.12)	0.04 (−0.13, 0.21)	0.03 (− 0.15, 0.20)
Wasting (<−2 z-scores)	40/1222 (3.3%)	26/791 (3.3%)	12/229 (5.2%)	78/2242 (3.5%)	1.02 (0.61, 1.71)	1.23 (0.59, 2.56)	1.20 (0.55, 2.62)
2016							
Length/height-for-age z-score	−0.71 (1.22)	− 0.61 (1.12)	− 0.83 (1.27) ^§^	−0.69 (1.20)	0.01 (− 0.09, 0.12)	0.06 (− 0.08, 0.20)	0.05 (− 0.09, 0.19)
Stunting (<−2 z-scores)	144/1078 (13.4%)	69/773 (8.9%) ^†^	51/332 (15.4%) ^§^	264/2183 (12.1%)	0.73 (0.53, 1.01)	0.99 (0.68, 1.43)	1.35 (0.89, 2.04)
Weight-for-age z-score	−0.31 (1.05)	− 0.19 (1.02)	− 0.32 (1.00) ^§^	−0.27 (1.03)	0.02 (− 0.07, 0.11)	0.09 (− 0.03, 0.21)	0.07 (− 0.06, 0.19)
Underweight (<−2 z-scores)	47/1086 (4.3%)	31/783 (4.0%)	10/335 (3.0%)	88/2204 (4.0%)	1.03 (0.63, 1.67)	0.52 (0.25, 1.08)	0.51 (0.24, 1.09)
Weight-for-length/height z-score	0.10 (0.96)	0.18 (1.03)	0.15 (0.88)	0.14 (0.97)	0.02 (−0.07, 0.11)	0.09 (−0.03, 0.21)	0.08 (− 0.05, 0.20)
Wasting (<−2 z-scores)	17/1073 (1.6%)	14/770 (1.8%)	2/328 (0.6%)	33/2171 (1.5%)	1.28 (0.61, 2.65)	0.38 (0.09, 1.73)	0.30 (0.07, 1.38)

*NLBC* non-left-behind children, *FLBC* left-behind children with migrant fathers, *PLBC* left-behind children with both migrant parents, *aMD* adjusted mean difference, *aOR* adjusted odds ratio

^a^The number of children with available outcomes was indicated with *N* after slash

^b^aMDs and aORs were adjusted for sociodemographic characteristics of children (resident county, child gender, age, preterm and ethnicity) in 2013, and additional two intervention variables (the frequency of consumption of Yingyangbao supplements and the frequency of received nutritional consultations) in 2016

^†^FLBC vs. NLBC *p* < 0.05; ^‡^ PLBC vs. NLBC *p* < 0.05; ^§^ PLBC vs. FLBC *p* < 0.05

In 2013, the results of univariate analyses show that PLBC had significantly lower WAZ (− 0.77 vs. -0.46, *p* < 0.01) and WLZ (− 0.24 vs. 0.10, *p* < 0.01) scores than NLBC, and also had significantly lower LAZ (− 1.07 vs. -0.57, *p* < 0.01), WAZ (− 0.77 vs. -0.19, *p* < 0.01), and WLZ (− 0.24 vs. 0.19, *p* < 0.01) scores and higher risks of stunting (18.6% vs. 11.8%, *p* < 0.01) and underweight (12.1% vs. 6.5%, *p* < 0.01) than FLBC; however, these differences were not statistically significant after controlling for children’s sociodemographic characteristics. In addition to the better performance in these anthropometric outcomes than PLBC aforementioned, FLBC were shown by univariate analyses to have significantly higher LAZ (− 0.57 vs. − 0.89, *p* < 0.01) and WAZ (− 0.19 vs. -0.46, *p* < 0.01) scores and lower risks of stunting (11.8% vs. 19.0%, *p* < 0.05) and underweight (6.5% vs. 9.2%, *p* < 0.05) than NLBC; even after controlling for children’s sociodemographic characteristics, their differences in LAZ scores (aMD 0.13 [95%CI: 0.01, 0.26]) and the risk of stunting (aOR 0.73 [95%CI 0.56, 0.96]) were significant (Table 2).

In 2016, both univariate and multivariate analyses identified no significant differences in the anthropometric outcomes between PLBC and NLBC. Although a lower risk of stunting (8.9% vs. 13.4%, *p* < 0.05) than NLBC and higher HAZ (− 0.83 vs. -0.61, *p* < 0.05) and WAZ (− 0.32 vs. -0.19, *p* < 0.05) scores than PLBC were found by univariate analyses among FLBC, no such differences were detected in the multivariable adjusted analyses (Table 2).

### Anemia

Table 3 shows the Hb concentration and the prevalence of anemia among children with various parental migration statuses. 44.8% of children in 2013 and 43.8% of children in 2016 were identified with anemia. A lower risk of anemia and higher Hb concentration was found by univariate analyses among PLBC compared with NLBC and FLBC both in 2013 (34.9% vs. 44.3 and 48.3%; 11.29 g/dL vs. 10.95 g/dL and 10.85 g/dL) and 2016 (35.2% vs. 45.0 and 45.9%; 11.15 g/dL vs. 10.87 g/dL and 10.80 g/dL). Multivariate logistic regressions reveal that the odds of anemia were significantly lower for PLBC than for FLBC (aOR 0.67 [95%CI 0.47, 0.94]) in 2013, and than for NLBC (aOR 0.74 [95%CI 0.56, 0.97]) and FLBC (aOR 0.75 [95%CI 0.47, 0.56, 1.00]) in 2016.

**Table 3:** Mean hemoglobin concentration and anemia prevalence among children aged 6–35 months with various parental migration statuses

	Outcomes [mean (SD)/ n/N (%)] ^a^				Adjusted differences [aMD (95%CI) / aOR (95%CI)] ^b^		
	NLBC	FLBC	PLBC	Total	FLBC vs. NLBC	PLBC vs. NLBC	PLBC vs. FLBC
2013							
Hemoglobin concentration (g/dL)							
6–17 months	10.43 (1.52)	10.38 (1.54)	10.99 (1.38) ^‡§^	10.44 (1.53)	−0.08 (− 0.29, 0.14)	**0.53 (0.10, 0.96)**	**0.60 (0.15, 1.04)**
18–35 months	11.30 (1.18)	11.23 (1.43)	11.38 (1.48)	11.29 (1.30)	−0.07 (− 0.22, 0.09)	0.05 (− 0.18, 0.29)	0.10 (− 0.15, 0.35)
Total	10.95 (1.39)	10.85 (1.54)	11.29 (1.46) ^‡§^	10.95 (1.46)	−0.08 (− 0.20, 0.05)	0.18 (− 0.03, 0.39)	**0.24 (0.02, 0.46)**
Anemia							
6–17 months	311/506 (61.5%)	229/360 (63.6%)	25/58 (43.1%) ^‡§^	565/924 (61.1%)	0.06 (−0.18, 0.30)	**0.75 (0.35, 1.15)**	**0.71 (0.29, 1.12)**
18–35 months	245/749 (32.7%)	160/445 (36.0%)	55/171 (32.2%)	460/1365 (33.7%)	−0.07 (− 0.27, 0.13)	−0.12 (− 0.36, 0.11)	−0.10 (− 0.35, 0.15)
Total	556/1255 (44.3%)	389/805 (48.3%)	80/229 (34.9%) ^‡§^	1025/2289 (44.8%)	1.17 (0.96, 1.41)	0.78 (0.56, 1.08)	**0.67 (0.47, 0.94)**
2016							
Hemoglobin concentration (g/dL)							
6–17 months	10.27 (1.59)	10.32 (1.88)	11.10 (1.04) ^‡§^	10.36 (1.68)	1.13 (0.84, 1.52)	**0.47 (0.27, 0.85)**	**0.42 (0.23, 0.76)**
18–35 months	11.31 (1.43)	11.14 (1.77)	11.17 (1.93)	11.23 (1.65)	1.17 (0.91, 1.52)	1.00 (0.67, 1.50)	0.85 (0.56, 1.31)
Total	10.87 (1.59)	10.80 (1.86)	11.15 (1.75) ^‡§^	10.89 (1.72)	−0.01 (−0.16, 0.14)	0.14 (−0.06, 0.34)	0.12 (−0.09, 0.33)
Anemia							
6–17 months	299/452 (66.2%)	193/321 (60.1%)	34/80 (42.5%) ^‡§^	526/853 (61.7%)	0.80 (0.58, 1.09)	**0.39 (0.23, 0.65)**	**0.49 (0.29, 0.82)**
18–35 months	187/628 (29.8%)	161/450 (35.8%) ^†^	83/252 (32.9%)	431/1330 (32.4%)	1.17 (0.89, 1.53)	1.00 (0.72, 1.39)	0.86 (0.61, 1.21)
Total	486/1080 (45.0%)	354/771 (45.9%)	117/332 (35.2%) ^‡§^	957/2183 (43.8%)	0.99 (0.80, 1.21)	**0.74 (0.56, 0.97)**	**0.75 (0.56, 1.00)**

*NLBC* non-left-behind children, *FLBC* left-behind children with migrant fathers, *PLBC* left-behind children with both migrant parents, *aMD* adjusted mean difference, *aOR* adjusted odds ratio

^a^The number of children with available outcomes was indicated with *N* after slash

^b^aMDs and aORs were adjusted for sociodemographic characteristics of children (resident county, child gender, age, preterm and ethnicity) in 2013, and additional two intervention variables (the frequency of consumption of Yingyangbao supplements and the frequency of received nutritional consultations) in 2016

^†^FLBC vs. NLBC *p* < 0.05; ^‡^ PLBC vs. NLBC *p* < 0.05; ^§^ PLBC vs. FLBC *p* < 0.05

Further, the results of stratified analysis for age group show that about one-third of children aged 18–35 months were anemic with no significant difference between children with different parental migration statuses (32.7% of NLBC, 36.0% of FLBC, and 32.2% of PLBC in 2013, and 29.8, 35.8, and 32.9% in 2016, respectively). However, in the children aged 6–17 months, about two-thirds of those living with mothers or both parents (61.5% of NLBC and 63.6% of FLBC in 2013, and 66.2% of NLBC and 60.1% of FLBC in 2016) were identified with anemia, which was much higher than the corresponding proportion in those with both migrant parents (43.1% in 2013 and 42.5% in 2016). Both univariate and multivariable adjusted analyses show that PLBC aged 6–17 months had a significantly higher Hb concentration and lower risk of anemia than NLBC and FLBC of the same age group in 2013 and 2016 (Table 3).

### Breastfeeding and dietary intake

**Fig. 1:**
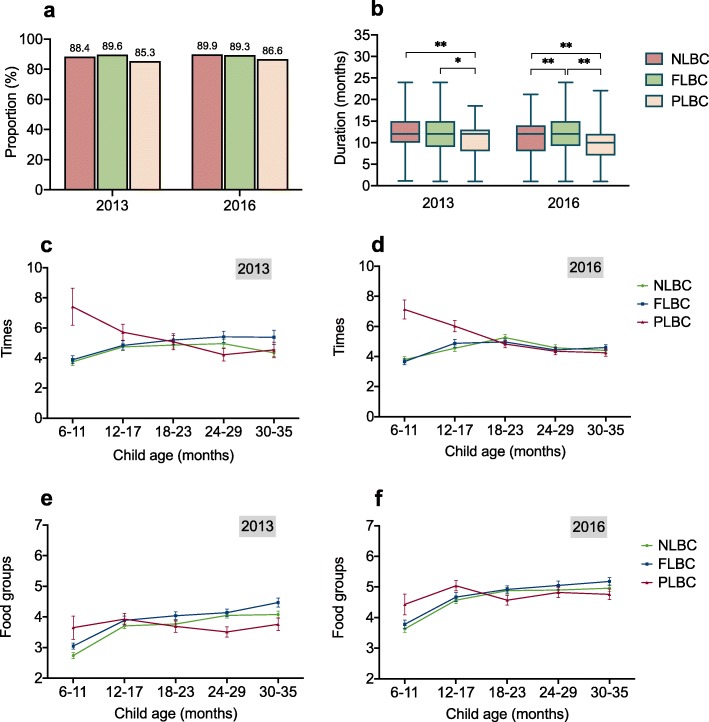
Breastfeeding and dietary intake of children with different parental migration status. NLBC, non-left-behind children; FLBC, left-behind children with migrant fathers; PLBC, left-behind children with both migrant parents. **a** Bar chart of the proportion of ever being breastfed. **b** Boxplots of breastfeeding duration; the central line in each box indicates the median, and the bottom and top edges of the box indicate the 25th and 75th percentiles respectively; the top and bottom whiskers extend to the 2.5th and 97.5th percentiles. **c** and **d** Line chart of meal frequency. **e** and **f** Line chart of dietary diversity. The symbols and bars on the line chart indicate the mean and the standard error of mean, respectively

Child breastfeeding and dietary intake are presented in Fig. 1. More than 85% of children were ever breastfed in 2013 and 2016, and the proportion was not significantly different between children with various parental migration statuses. PLBC had a shorter duration of breastfeeding than FLBC and NLBC (10.82 vs. 11.89 and 12.26 months in 2013, and 9.98 vs. 12.28 and 11.31 months in 2016). The median duration of breastfeeding in FLBC was similar to NLBC in 2013 but longer than NLBC in 2016. Children’s dietary diversity increased from 2013 to 2016, irrespective of their parental migration status. In 2013, FLBC had slightly higher meal frequency and dietary diversity than NLBC, but in 2016, the differences were of lesser magnitude or absent. Compared with NLBC and FLBC, PLBC had higher meal frequency and dietary diversity at age of 6–17 months but slightly lower meal frequency and dietary diversity at age of 18–35 months.

## Discussion

Early childhood nutrition is important as it lays the foundation for health in later life. In the context of rapid development and large-scale rural-to-urban migration in China, we evaluated the early childhood nutrition of rural left-behind children using the latest and reliable data. Our work not only extends the observations to children of younger age but also improves our understanding of the association between various parental migration statuses and child nutrition in the current social context.

The findings demonstrate the improvement of early childhood nutrition in recent years in rural China. The estimated risks of stunting, underweight, and wasting among rural infants and toddlers in 2013 in this study are consistent with previous reports during the similar period [10, 15, 16]. A study using the data of 6136 children aged 0–3 years in central and western China during 2010–2011 showed that the prevalence of stunting among NLBC, FLBC, and PLBC was 16.4, 15.1, and 16.6%, respectively [15]. A national survey of the nutritional status of rural left-behind children under 7 years old was conducted among 7585 left-behind children and 7557 non-left-behind children from 13 provinces of China during 2008–2009, and found that the prevalence of stunting among under-7-year-old children with non-migrant, one, and both migrant parents was 16.3, 14.9, and 17.9%, that of underweight was 7.6, 7.2, and 8.3%, and the prevalence of wasting was 3.3, 3.1, and 3.4%, respectively [16]. However, these estimates are lower than the corresponding values derived from our survey in 2016. In 2016, the estimated prevalence of stunting, underweight and wasting among surveyed children aged 6–35 months were 12.1, 4.0, and 1.5%, respectively, which decreased by 26.2, 52.9, and 57.1% compared with 2013, respectively, and are lower than other developing countries [19]. Child dietary diversity had also been greatly improved, irrespective of their parental migration status.

Consistent with previous estimates [10, 28, 29], this study also highlights the constantly and alarmingly high risk of anemia among children under 3 years old in rural China. The results show that about two-thirds of children aged 6–17 months and one-third of the children aged 18–35 months were anemic, and no significant reduction of risk of anemia was found among these children from 2013 to 2016. Children aged 6–17 months with migrant parents had a significantly lower risk of anemia compared with those living with their mothers or with both parents, which may be attributed to earlier breastfeeding cessation and complementary feeding because of maternal migration. Despite the benefits of breastfeeding to child growth and development, prolonged breastfeeding was found to be associated with decreased dietary intakes and increased risk of anemia in infants and young children over 6 months of age [30–34]. This association was not significant at ≥18 months of age when most children are weaned from breastfeeding irrespective of the parental migration status. The results also suggest the critical problem in complementary feeding among rural children. Most mothers and caregivers may not have good knowledge about and practices in breastfeeding and complementary feeding [29, 35].

The present study broadly supports the previous findings that parental migration was not significantly detrimental to child nutrition [13, 16, 22, 36] and even confirm the benefits of paternal migration on early childhood nutrition of rural children [13, 37, 38], despite contrary results found in other studies [5, 18, 39]. A decreasing difference in early childhood nutrition between children with various parental migration statuses was shown in recent years, and in the survey in 2016, no significant differences in risks of stunting, underweight, and wasting were detected between them. Except for the obvious substitution of complementary foods for breast milk among children with migrant parents before 18 months of age, the difference in dietary intake among children with different parental migration statuses also decreased. However, it is worth noting that our findings do not mean that children in rural China are well-nourished or that left-behind children are not vulnerable. Despite the great reduction in the risk of macronutrient deficiency, all rural children were still at high risk of micronutrient deficiency such as anemia. In addition, a growing body of literature highlights the need for special attention to the negative effects of parental migration on children’s cognitive and social-emotional development, especially when both parents migrate [40–42].

The mechanism by which parental migration affects child nutrition may be very complex. On the one hand, migration brings economic benefits, broadens the families’ horizons and leads to new life perspectives, which is beneficial to the health of children by increasing health-related investment, improving left-behind caregivers’ mental health, and substantially changed parenting attitude and practices [13, 22, 43, 44]. On the other hand, the absence of one or both parents can increase psychological stress among left-behind children as well as caregivers [45–49]; it can also reduce the time allocated to child care within households and even causes caregiver rearrangement and changed feeding practices when both parents migrate [50]. These two effects are working against one another, leading to the indeterminate net effect of migration on early childhood nutrition. In our study, FLBC had better household economic status than NLBC in 2013, which may partly account for the lower risk of undernutrition among FLBC; in 2016, they had similar household economic status, and the differences in the risk of undernutrition between them were also of lesser magnitude than those in 2013. However, we found families with migrant parents may be poorer than families with non-migrant parents or with migrant fathers only. This may imply a complex relationship between labor migration and family economics: migration of more family members brings more economic gains but also may indicate poorer family economics. Caregivers’ education and depressive symptoms affect child nutrition mostly by affecting feeding practices [51–54]. In our study, compared to caregivers included in 2013, those included in 2016 had significantly higher levels of education and less depressive symptoms, which may partly explain the improvement of feeding practices and child nutrition during this period. Increasingly similar determinants between children with various parental migration statuses may also account for their decreasing differences in nutritional outcomes in recent years. It is worth noting that rural areas in China have developed rapidly in recent years, which may greatly contribute to the improvement of child nutrition. Overall, a variety of healthy foods are increasingly available in rural areas, and farmers are getting richer. Even with limited income, many farmers have improved water, hygiene and sanitation, and renovated their houses with the support of the government. Increasingly convenient transportation, mailing and communication technology also make it easier for migrant parents to participate in child feeding.

Our study has implications for the formulation of programs and policies to improve child health and development in rural China. The results show a high risk of anemia among rural children, regardless of parental migration status, suggesting a broad need for nutritional support for all rural children, especially prevention from micronutrient deficiency. Mothers are suggested to accompany their children in early childhood given the important health benefits of breastfeeding. Nutrition education and some micronutrient fortified foods can be provided to all caregivers. The Chinese government provides special social services for left-behind children as one of the goals of the National Program of Action for Child Development in China (2011–2020) [55]. However, our findings indicate that due to social and economic development, left-behind children may have equal or slightly better nutrition than non-left-behind children. Therefore, programs and policies designed to promote the well-being of left-behind children should pay more attention to other important issues such as cognitive and social-emotional development.

Our study has some strengths. Repeated cross-sectional data of two large-sample surveys in the same areas were used. In each survey, well-established and validated methods were employed to measure child nutritional status by investigators blinded to parental migration status and the basic characteristics of the children and their caregivers. However, several limitations also exist. First, the low response rate may bias the results. About 40% of the eligible children in the selected villages were not enrolled in the surveys. According to rough interviews with local coordinators, the reasons why the caregivers with their children did not participate in the surveys probably include being busy with household tasks, lack of interest or being not at home, etc. Second, we failed to measure some important factors that may moderate the effects of parental migration on the health of left-behind children, such as the number of siblings, migration duration, the age of children at the time of the first separation from migrant parents, migrant-caregiver communication, and remittances from migrants [36, 48, 56–58]. Third, the interventions of the early childhood development program may confound the analysis of the 2016 survey data, but we controlled for related variables in the regression analyses. Fourth, this study is also limited by its cross-sectional design, and the resulting associations need to be further demonstrated by longitudinal studies. Finally, the generalizability of the results is limited because all participants were from poverty-stricken rural areas of northern and southern China, and about half of them were from three counties of Guizhou Province where a large number of residents are of minorities. In addition, in rural China, many villages have less than 50 children under 3 years of age, which means the selected villages may not be that representative.

## Conclusions

In conclusion, this study provides information on the changes over time of early childhood nutrition of left-behind children in rural China, and provides new evidence that parental migration is no longer detrimental and even beneficial to the nutrition of these children but all the children are still at high risk of anemia. Although future programs for left-behind children should continue to focus on nutrition, they should also pay more attention to other important health issues such as mental health. Further research is needed to develop a clearer and full-scale picture of the impact of parental migration on the well-being of left-behind children of all ages.

## Data Availability

The datasets used and/or analysed during the current study are available from the corresponding author on reasonable request.

## Abbreviations

aMD: adjusted mean difference
aOR: adjusted odds ratio
FLBC: Left-behind children with migrant fathers
Hb: Hemoglobin
LAZ: Length-for-age Z score
LBC: Left-behind children
NLBC: Non-left-behind children
PLBC: Left-behind children with both migrant parents
WAZ: Weight-for-age Z score
WHO: World Health Organization
WLZ: Weight-for-length Z score
ZSDS: Zung’s self-rating depression scale
